# Supplementary material for: A Novel Cystatin Gene from Sea Cucumber (Apostichopus japonicus): Characterization and Comparative Expression with Cathepsin L During Early Stage of Hypoxic Exposure-Induced Autolysis
Source: Foods. 2025 Apr 18;14(8):1404. doi: 10.3390/foods14081404 (PMC12027202; doi:10.3390/foods14081404)
Supplement: Supplementary file 1 [file foods-14-01404-s001.zip › foods-3518289-supplementary.pdf]

Amino acid sequences and accession numbers of 22 species in the developmental tree

1>cystatin A [Lepeophtheirus salmonis] （ADD38609）

MMVGGIGVLEQATPEVQEIIDGLKPKIEEKIGKSVDKLTLISFKTQVVAGRNYFAKVKAGDNDHIHVRIY
HDLSNAKTLTSVQTEKSHEDEIEYF

2>cystatin A[Mus musculus]（AAI39157）

MIPGGLTEARPATAEVQEIADRVKAQLEEETNEKYEIFKAVEYKTQVVAGVNYFIKMDVGGGCFTHIKV
FKDLSGKNNLELTGYQTNKTEDDELTYF

3>cystatin A [Homo sapiens]（AAH10379）

MIPGGLSEAKPATPEIQEIVDKVKPQLEEKTNETYGKLEAVQYKTQVVAGTNYYIKVRAGDNKYMHLK
VFKSLPGQNEDLVLTGYQVDKNKDDELTGF

4>cystatin B [Ruditapes philippinarum]（AFP50149）

MCGGAGDVMPADEEVKGYCNEVKADILKKAGKDSVEIFEPVHYRSQLVAGVNYFVKIRIGSGGECLH
ARIFKGLPHTGGNLEVSSVQTNKKVEDAVEYF

5>cystatin B [Psetta maxima]（ADM61584）

MPLCGGLGESANADDDIQKICDSMKPHAEEKTGKSFAVFTAKTYKTQLVSGTNYFIKVHVGEEEHLHI
RVYKKLQCNGGEIELTSLQENKSHHDPIVYF

6>cystatin B [Perinereis aibuhitensis]（ACL12062）

MMSGGLGPTLPADPETQVICDEVKNQLEGEVGRNFAEYNAILFRSQVVAGTVLFIKVHVGHADYIHIR
IFIPLPGPGKTSLGGYQLHKTKDDPIKYFNNN

7>cystatin B [Sus scrofa]（ABN71232）

MMCGAPSATQPATAEIQAIADKVKSQLEEKENKTFPVFKAVEFKSQVVAGRNLFIKVQVDDDNFVHL
RVFESLPHENKPLTLSSYQTNKSRHDELTYF

8>cystatin B [Gadus morhua]（AEK21704）

MPMMCGGTTPDKPATKEVQDLCDTVKASVEAKANKSYEVFMAKTYKTQVVAGTNFFIKVHVGGED
HVHLRVFRELPCNGGNVKLSDMQESKSHQDPLEHF

9>cystatin B [Oncorhynchus mykiss]（NP_001117971）

MKCGGTAEAKDATPEVQQICDEMKPYAEEKAAKKFDVFAAKTFTTQVVAGTNFFIKVHVGGDEFVHL
RVHRTLPHAGGKLELHGVQTSKAHNDPIGFF

>10 Cystatin-1(cystatin-like) [Chilobrachys jingzhao] (B1P1J3)

MKAIYLILTVLCGFSASTKTGGWRDKDVDDEDIRKFATLAASENSKMSNSLYFEKLVKVI
EAKSQVVSGVKYNITFEIAPTECKKNGKGYDKLSECPLLESAPHQTCTAIIWTRSWLNDT
QILKLKCKEGGSSC

11>cystatin C [Paralichthys olivaceus]

MKMLVFPVLAALFAVGLGNLVGAPRDINISEAQDALDFAVAKHNSGTNDMFLRQVAEVVRVQRQV
VSGNKYIITVKMAKTPCRKDRVVNEVCEIHKDPALAQPYECTFSVWSRPWIPDLQLVGEKC

12>cystatin C [Rattus norvegicus]（NP_036969）

MASPLRSLMLLLAVLAVAWAGTSRPPPRLLGAPQEADASEEGVQRALDFAVSEYNKGSNDAYHSRAI
QVVRARKQLVAGINYYLDVEMGRTTCTKSQTNLTNCPFHDQPHLMRKALCSFQIYSVPWKGTHTLT
KSSCKNA

13>cystatin C [Ovis aries]（AFV58072）

MVGSPRAPLLLLAALIVSLALALSPVAAQGPRKGRLLGGLMEADVNEEGVQEALSFAVSEFNKRSNDA
YQSRAMRVVRARKQVVSGMNYFLDVKLGRTTCTKSQTNLDSCPFHDQPHLKREKLCSFQVYVVPW
MNTINLVKFSCQD

14>cystatin C [Macaca mulatta]（AAW79565）

MAGPLRAPLLLLAILAVALAVSPAAGASPGKPPRLVGGPMDASVEEEGVRRALDFAVSEYNKASNDM
YHSRALQVVRARKQIVAGVNYFLDVELGRTTCTKTQPNLDNCPFHEQPHLKRKAFCSFQIYTVPWQG
TMTLSKSTCQDA

15>cystatin S [Homo sapiens]（AAH74952）

MARPLCTLLLLMATLAGALASSSKEENRIIPGGIYDADLNDEWVQRALHFAISEYNKATEDEYYRRPLQ
VLRAREQTFGGVNYFFDVEVGRTICTKSQPNLDTCAFHEQPELQKKQLCSFEIYEVPWEDRMSLVNSR
CQEA

16>cystatin SN [Homo sapiens]（AAH21225）

MAQYLSTLLLLLATLAVALAWSPKEEDRIIPGGIYNADLNDEWVQRALHFAISEYNKATKDDYYRRPLR
VLRARQQTVGGVNYFFDVEVGRTICTKSQPNLDTCAFHEQPELQKKQLCSFEIYEVPWENRRSLVKSR
CQES

17>kininogen[Danio rerio]（AAH83429）

MARDKILTVLAMLWLYFCGGLAQTDSSVPCDDRRVEKVVNLTLGTHNKMITEGAQLALYEILEATKA
QNESGDVLLVRFSSRETDCPAGGEKTWHECDYLQQADKALRICHAKVQFTEAGEELLLHDCLEPAIIA
SVAPCLGCPENIDVHKEELRQPLIHSLSKANSMINHVHFFIFKDLTSATKQVVAGFRYKLQFEIEKSNCT
RPEFKIVTEECHPLLEKTEVLKCNSSVDVAPWRHEVPEVHVVCEAGVSKTNSRFKRPPGWSPLRMLPQ
AKESSEESKESISPPKHVPLNCPTKPWKEFKPIIAPPNATEPSEPSADTALSDLDLIR

18>kininogen[Carassius auratus]（AGO58862）

YECDYLQQTDKAIINCQAKVQFEEADQKLLLHDCTAEPAASSKVAQCLGCPEKIDLHHKELKEPLIYSL
SKANSIVKHNHFFIFRHLTFATKQVVAGFRYKLQFLIEKSNCTRAEFKVVTEECHPMQEKTEVLQCNST
VDVAPWRHERPEVHVECETMVTKSVARFKRPPGWSPIRKLKPLNCPSIPWRE

19>kininogen[Homo sapiens]（AAH60039）

MKLITILFLCSRLLLSLTQESQSEEIDCNDKDLFKAVDAALKKYNSQNQSNNQFVLYRITEATKTVGSDT
FYSFKYEIKEGDCPVQSGKTWQDCEYKDAAKAATGECTATVGKRSSTKFSVATQTCQITPAEGPVVTA
QYDCLGCVHPISTQSPDLEPILRHGIQYFNNNTQHSSLFMLNEVKRAQRQVVAGLNFRMTYSIVQTN
CSKENFLFLTPDCKSLWNGDTGECTDNAYIDIQLRIASFSQNCDIYPGKDFVQPPTKICVGCPRDIPTN
SPELEETLTHTITKLNAENNATFYFKIDNVKKARVQVVAGKKYFIDFVARETTCSKESNEELTESCETKKL
GQSLDCNAEVYVVPWEKKIYPTVNCQPLGMISLMKRPPGFSPFRSSRIGEIKEETTSHLRSCEYKGRPPK
AGAEPASEREVS

20>cystatin-like[Homo sapiens]（AAI17397）

MGIGCWRNPLLLLIALVLSAKLGHFQRWEGFQQKLMSKKNMNSTLNFFIQSYNNASNDTYLYRVQR
LIRSQMQLTTGVEYIVTVKIGRTKCKRNDTSNSSCPLQSKKLRKSLICESLIYTMPWINYFQLWNNSCLE
AEHVGRNLR

21>cystatin-like[Latrodectus hesperus]（ADV40303）

MGVSLKILICLSMLCLAASMMTGGWQKELNPNSESIQKYAKFATAKVSASSNSLHHLKLTRISNVEKQ
VVSGMNYKMTITMAPTECKKNGNSTQLNIDECPLLKCGAPNICNVTVWVQAWMENGIKLTKSSCT
QGVSAC

22> Cystatin domain-containing protein (cystatin-C-like) [Patiria miniata (bat star)] (XP_038074123)

MSSLLLLTGVFLVSLVTLSASSGLGGLEPAKVDEAGVLRSAHFAMGQINKKSNALYASKM
TKIINAQKQVVSGMNYYLTIETTETECKNTGPIDDLDSCELLDKPKKQICEVVVNEELWM
KENPRKLLDSSCQ
